# Sepsis in hemodialysis patients

**DOI:** 10.1186/s12873-015-0057-y

**Published:** 2015-10-14

**Authors:** Gilbert Abou Dagher, Elie Harmouche, Elsy Jabbour, Rana Bachir, Dina Zebian, Ralphe Bou Chebl

**Affiliations:** Department of Emergency Medicine, American University of Beirut, Beirut, Lebanon; Department of Emergency Medicine, Henry Ford Hospital, Detroit, Michigan USA

**Keywords:** Sepsis, Dialysis, Bacterial, Mortality, Emergency management

## Abstract

**Background:**

Bacterial infections are very common in End Stage Renal Disease (ESRD) patients. The diagnosis of sepsis in such patients is often challenging and requires a high index of suspicion. The aim of this study is to report on a series of patient with ESRD on hemodialysis (HD) diagnosed with sepsis.

**Methods:**

Single center retrospective study looking at ESRD on HD who presented to our tertiary hospital were retrieved. Inclusion criteria included a discharge diagnosis of sepsis, septic shock or bacteremia.

**Results:**

Our sample was composed of 41 females and 49 males, with a mean age of 70 ± 15 years. Infections from the HD catheters followed by lower respiratory tract infections were the most common cause of bacteremia. IV fluid replacement for the first 6 and 24 h were 0.58 and 1.27 l respectively. Vasopressors were used in 30 patients with norepinephrine, dopamine and dobutamine used in 22, nine and one patients respectively. Out of 90 subjects, 24 (26.6 %) were dead within the same hospital visit. the 28 days out of hospital mortality was 25.6 %. There was no significant difference in mortality in patients who presented with less than two SIRS or two or more SIRS criteria.

**Conclusion:**

This is the first study looking at an in depth analysis of sepsis in the specific dialysis population and examining the influence of fluid resuscitation, role of SIRS criteria and vasopressor use on their mortality.

## Background

Sepsis ranks as the 10th leading cause of death in the United States. Rivers et Al. showed that early identification of sepsis and implementation of Early Goal Directed Therapy (EGDT) have been shown to improve outcomes and decrease mortality in patients with severe sepsis and septic shock [1]. Consequently, hospitals have implemented protocols and sepsis bundles in order to decrease its associated mortality and morbidity [2, 3]. Sepsis and bacterial infections are very common in End Stage Renal Disease (ESRD) patients [4–6] and following cardiovascular disease; infection is the second leading cause of death in patients with ESRD [6, 7]. Emergency Physicians are often faced with the hypotensive, weak ESRD patient presenting from dialysis and are usually anchored on the diagnosis of hypovolemia. The diagnosis of sepsis in such patients is often challenging and requires a high index of suspicion. Most studies on sepsis, ranging from the sensitivity of the systematic inflammatory response syndrome (SIRS) criteria [8] to lactate clearance [9] and optimal fluid therapy have looked at a general patient population. The aim of this study is to report on a series of patients with ESRD on hemodialysis (HD) diagnosed with sepsis, and to determine whether initial presentation, comorbidities, time to antibiotics or resuscitation, disposition affects hospital and 1 month mortality.

## Summary

The aim of this study is to report on a series of hemodialysis patients admitted to the hospital with a diagnosis of sepsis or septic shock. We looked at their in hospital mortality, at the parameters of resuscitation and at the value of the SIRS criteria. As such, this article sheds light on a special subset of population and should be of interest to nephrologists.

## Methods

This was a retrospective study approved by our Institutional Review Board (IRB# ER.GA.03). Patients with a known history of ESRD on HD presenting to the Emergency Department at a single university based institution within a 5-year period were retrieved. Patients were included if the discharge diagnosis was sepsis, bacteremia or septic shock. Patients younger than 18 years old, pregnant, presenting secondary to trauma or with incomplete documentation were excluded. Age, gender, ethnicity, history of diabetes, hypertension, cardiac disease, cerebrovascular accidents, dyslipidemia, immunosuppression, malignancy and presence of other comorbidities were obtained from subjects’ medical record. Patients’ presentation including temperature, heart rate, respiratory rate, oxygen saturation, blood pressure and number of SIRS criteria were collected at initial presentation to the Emergency Department. Site of infection, causative microorganism and presence of bacteremia were retrieved as well as complete blood count, electrolytes, lactate, cardiac enzymes and arterial blood gas results and coagulation profile results. Time to antibiotics and amount of fluid resuscitation within the first 6 and 24 h, duration and type of vasopressors and steroids administration was noted. Disposition from the emergency department (ED), length of stay (LOS) in the ED, intensive care unit (ICU) or general practice unit (GPU) was calculated. Hospital mortality was noted as well as 28-day mortality.

## Statistical analyses

A two tailed sample *t*-test compared the difference in mean age, LOS in ED, ICU or GPU, LOS in hospital, time to and duration of vasopressors, antibiotics and steroids, fluid replacement at 6 and 24 h, vital sings at presentation and 6 h, electrolytes and blood work between deceased patients and non-deceased. Pearson’s chi-squared test compared difference in distribution of bacteremia, comorbidities, microbiology, disposition from the ED, use of vasopressors or steroids, location where antibiotics were started, number of SIRS criteria at presentation between deceased subjects and non-deceased. Statistical analyses were performed using SPSS Statistics for Windows Version 21.0. (Armonk, NY: IBM Corp).

## Results

### Demographics

Our sample was composed of 41 females and 49 males, with a mean age of 70 ± 15 years. Baseline characteristics at presentation are listed in Table 1.

**Table 1:** Baseline characteristics of the patients

Age (yr)	69.6 ± 15
Sex (%)	
Female	45.6
Male	54.4
Chronic coexisting conditions (%)^a^	
Hypertension	83.3
Diabetes	63.3
Coronary artery disease	45.6
Dyslipidemia	31.5
Congestive heart failure	34.5
Malignancy	11.1
Chronic Obstructive lung disease or emphysema	10
Neurologic disease	7.7
Liver disease	4.5
SIRS criteria at presentation (%)	
More than 2	70
Less than 2	30
Vital signs upon presentation	
Temperature (°C)	37.4 ± 1.1
Systolic Blood Pressure (mm Hg)	116 ± 31.7
Diastolic Blood Pressure (mm Hg)	61.1 ± 15.5
Mean Arterial Pressure (mm Hg)	80.0 ± 19.2
Heart Rate (beats/min)	86.2 ± 19.4
Respiratory Rate (respiration/min)	21.2 ± 5.6
Oxygen Saturation (%)	96.0 ± 5.0
Base line laboratory values	
White-cell count (per mm3)	14 ± 7*10000
Hemoglobin	10.9 ± 1.97
Hematocrit	32.8 ± 6.1
Lactate (mmol/liter)	29 ± 4.1
Lactate ≥4 (%)	81.3
Lactate ≤4 (%)	18.8
Creatinine (mg/dl)	5.4 ± 2.3
Blood urea nitrogen (mg/dl)	51.5 ± 24.5
Total bilirubin (mg/dl)	0.54 ± 0.6
Partial pressure of carbon dioxide (mm Hg)	35.2 ± 16.1
pH arterial blood	7.38 ± 0.01
PaO2/FiO2	318.7 ± 141.6
Bacteremia	
Yes	39
No	51
Site of Infection (%)	
HD Catheter	26.7
Unknown	20.0
Lung	15.6
Urine	11.1
Skin	11.1
GI	8.9
Gall Bladder	3.3
Brain	1.1
Heart	1.1
Bone	1.1

^a^Values sum to more than 100 % because patients could have more than one condition

### Management

Out of 90 patients, 39 were bacteremic. Infections from the HD catheters followed by lower respiratory tract infections were the most common cause of bacteremia (Table 1). *Escherichia coli* followed by *staphylococcus coagulase negative* were responsible for the majority of infections (Table 2).

**Table 2:** Causative microorganisms

Microbiology (N, %)	
E. coli	22 (24.4 %)
Staphylococcus coagulase negative	20 (22.2 %)
Klebsiella pneumonia	7 (7.8 %)
Pseudomonas aeruginosa	7 (7.8 %)
Enterococcus species	6 (6.7 %)
Staphylococcus aureus	5 (5.6 %)
Candida species	5 (5.5 %)
Proteus mirabilis	4 (4.4 %)
Serratia species	3 (3.3 %)
Acinetobacter baumani	2 (2.2 %)
Bacteroid fragilis	2 (2.2 %)
Enterobacter cloacae	2 (2.2 %)
Others	6 (6.6 %)
*Clostridium species, Listeria monocytogenes, Diphteroid species, Morganella morganii, Brucella species, Providencia alcaligenese*	

IV fluid replacement for the first 6 and 24 h were 0.58 and 1.27 l respectively. Vasopressors and inotropes were used in 30 patients with norepinephrine, dopamine and dobutamine used in 22, 9 and 1 patients respectively. Mean time to vasopressors and duration of use were 8.41 ± 7 h and 5.86 ± 7.0 days respectively. Steroids were used in 23 patients. In 78 patients, antibiotics were initiated in the ED, one in the ICU and 11 on the general practice unit. Mean time to antibiotics administration was 4.47 ± 7.6 h (Table 3).

**Table 3:** Patient management characteristics

	Number	Mean ± SD	Range
Length of Stay in ED^a^ (Hours)	90	14.05 ± 13.188	2.00–87.75
Length of stay in ICU^a^ (Days)	24	8.88 ± 16.346	0.04–81.00
Length of stay in GPU^a^ (Days)	66	6.06 ± 5.179	0.13–32.88
Length of stay in the Hospital (days)	90	9.20 ± 9.981	1.04–81.08
Time to vasopressors in the first 24 h (hours)	13	8.41 ± 7.026	1.13–23.16
Time to Levophed (hours)	22	62.56 ± 85.096	1.13–315.00
Vasopressors therapy duration (days) for those who took vasopressors in the first 24 h	13	5.68 ± 6.989	0.33–22.50
Steroid Therapy duration (days)	23	14.70 ± 20.863	1.00–85.00
Time to Antibiotics treatment initiation (Hours)	89	4.47 ± 7.605	0.17–55.50
Intravenous fluids requirement first 6 h (Liters)	90	0.58 ± 0.827	0.01–5.00
Intravenous fluids requirement first 24 h (Liters)	90	1.27 ± 1.306	0.20–6.67

^a^ *ED* Emergency Department, *ICU* Intensive Care Unit, *GPU* General Practice Unit

### Disposition

Mean length of stay in the ED, ICU and GPU was 0.58 ± 0.55, 8.88 ± 16.3 and 6.1 ± 5.2 days respectively. 24 out of 90 were admitted to the ICU unit prior to being admitted to regular general floor. Out of 90 patients, 24 (26.6 %) died within the same hospital visit, 12 in the ICU and 12 on regular floor. There was no death in the ED. Out of 65 discharged subjects; the 28 day out of hospital mortality was 25.6 % (Table 4).

**Table 4:** Disposition of septic ESRD patients

	Number	Percent
Admission		
ICU^a^	24	26.7 %
GPU^a^	66	73.3 %
Hospital Mortality	24	26.7 %
Discharge Home	66	73.3 %
Mortality 28 days		
Yes	23	25.6 %
Unknown	4	4.4 %

^a^ *iCU* Intensive Care Unit, *GPU* General Practice Unit

### In hospital mortality

Total in hospital mortality was 24 (26.7 %). The in hospital mortality of septic shock patients was 40 %. There was no significant difference in mean age, gender distribution and comorbidities between the discharged and deceased group. Deceased patients presented with a higher mean heart rate (95.4 ± 25 versus 82.4 ± 15, *p* = 0.015), had a lower serum bicarbonate values (18.25 ± 5 versus 21.3 ± 3.5, *p* = 0.009) and had a significantly higher fluid requirements within the first 24 h (2.05 ± 1.7 L versus 0.98 ± 0.98 L, *p* = 0.008). There was no significant difference in mortality in patients who presented with less than two SIRS or two or more SIRS criteria. Thirty-three patients who were discharged had positive blood cultures compared to 18 from the deceased group (*p* = 0.034) however there was no significant difference in microbiology between the two groups (Table 5).

**Table 5:** Effect of treatment variables on ESRD patient mortality

	Mortality (No)		Mortality (Yes)		p-value
	N	%	N	%	
Levophed	12	54.5 %	10	45.5 %	0.022
Dopamine	3	33.3 %	6	66.7 %	0.004
Steroids use	12	52.2 %	11	47.8 %	0.008
Antibiotics Initiated in ED^a^	55	71.4 %	22	28.6 %	0.320
Antibiotics Initiated in ICU^a^	0	0 %	1	100 %	0.015
Antibiotics Initiated in GPU^a^	10	90.9 %	1	9.1 %	0.140
SIRS^a^					
0 or 1	20	74.1 %	7	25.9 %	0.917
≥2	46	73.0 %	17	27.0 %	
Bacteremia					
No	33	64.7 %	18	35.3 %	
Yes	33	84.6 %	6	15.4 %	0.034
ICU^a^ Admission	12	50.0 %	12	50.0 %	0.003
GPU^a^ Admission	54	81.8 %	12	18.2 %	0.003

^a^ *ED* Emergency Department, *ICU* Intensive Care Unit, *GPU* General Practice unit, *SIRS* Systemic Inflammatory Response Syndrome

Deceased patients were more likely to be admitted to the ICU compared to discharged patients (*p* = 0.003). Patients who received norepinephrine, dopamine or steroids had a significantly higher mortality than the discharged subjects (*p* = 0.022, 0.004 and 0.008 respectively) (Table 5).

## Discussion

Since Early Goal Directed Therapy (EGDT) by Rivers et al. was published there has been a steady decrease in sepsis related mortality [2, 3]. The rate of hospitalizations due to severe sepsis however has doubled during the past decade with estimates indicating that approximately 750,000 persons are affected annually in the USA [10]. Although much of the therapy for severe sepsis occurs in intensive care units (ICU), as many as 500,000 cases of severe sepsis are initially managed in emergency departments (ED) annually, with an average ED length of stay of 5 h [11–13].

Hemodialysis patients are at risk for infections. Using the US Renal Data System, Powe et al. aimed to look at the incidence of infections in dialysis patients and found that during 7 years of follow-up, 11.7 % of all hemodialysis patients and 9.4 % of peritoneal dialysis patients had at least one episode of septicemia [14]. To the best of our knowledge, there is one study that looked at sepsis related mortality in ESRD patients. Sarnak and Jaber examined dialysis patients death registry and compared sepsis related mortality to that of the general population and found that sepsis mortality was 100- to 300-times higher for chronic dialysis patients than the general public [6]. Hypothesized reasons for this association include increased susceptibility to infection, the presence of comorbidities such as diabetes, and repetitive exposure to pathogens during hemodialysis [15]. Our study is unique in that it is one of the fewest studies to look at sepsis solely in ESRD patients. We tried to look at the in hospital mortality of sepsis in ESRD, and also tried to look at the diagnostic value of SIRS and lactate in this subset of population. Similar to previous studies the most common source of infection arises from indwelling catheters followed by lower respiratory tract infections [14]. The most common organism causing sepsis was the gram negative Escherichia Coli followed by the skin colonizer Staphylococcus Epidermidis (Table 2).

The in hospital mortality for ESRD patients in septic shock remains at a staggering 40 %. Septic shock is one the leaders in mortality nowadays but with implementation of EGDT and early recognition and treatment, we have seen an improvement in septic shock mortality [16]. The mortality from septic shock was found to be as high as 46 % in the original EGDT control group but the implementation of protocols aiming at early identification and aggressive care has lead to an improved survival and presently the mortality from septic shock ranges between 20 and 30 [1, 16, 17]. Recent research has shown that the most important and cornerstone of sepsis therapy is early recognition, aggressive fluid hydration and early antibiotics [18, 19].

The out of hospital 28-day mortality of any ESRD patient admitted for sepsis remains at 25 %. ESRD patients are predisposed to infections because of the presence of invasive lines but are also unable to fight invading organisms because of impaired phagocytic function [20]. This raises the issue that even after they complete their course of antibiotics, they are still at a very high risk of death and need very close monitoring in the post discharge period.

Fluid resuscitation is the cornerstone of sepsis management [1, 18, 19]. The surviving sepsis campaign recommends a 30 cc/kg bolus in the first 3 h of resuscitation [16]. Further fluid therapy is guided by invasive hemodynamic monitoring such as the central venous pressure. Dialysis patients often have a complex presentation to the ED, while they appear fluid overloaded on examination; they often are hypotensive from being intravascularly depleted. Clinicians are often anxious to aggressively hydrate dialysis patients in efforts to avoid exacerbating their volume status. Such as demonstrated by our study, our ESRD patients were severely under resuscitated and there was a delay in antibiotic initiation, two elements that might have contributed to the elevated mortality. There is a shifting paradigm in the treatment of sepsis currently, notably moving away from invasive monitoring and focusing on early aggressive hydration and antibiotics. We believe that this also applies to ESRD patients, and emphasis should be on these two points with intubation indicated if there is an exacerbation of their volume status or respiratory distress arises. Excess fluid can always be removed by dialysis once the sepsis and bacteremia episodes are resolved.

The SIRS criteria were proposed as a screening method to rapidly flag possible septic patients, as sepsis is defined as having two or more SIRS criteria in the setting of a presumed or documented infection. Even though they were sensitive, SIRS criteria were not specific and did not correlate with mortality [8]. Owing to the fact that our study was a retrospective one, we selected patients based on their discharge diagnosis and wanted to look at the value of the SIRS criteria at presentation. It is interesting that in our study, patient who presented with less than two SIRS criteria had the same mortality as patients having two or more criteria. Emergency physicians should therefore be aware of this and should maintain a high level of suspicion when caring for ESRD patients as sepsis can present with normal vital signs.

This is a retrospective study done in a single emergency department and as such our study has many limitations. Some of the patients did not have repeat vitals at 6 h, and lactate wasn’t drawn on all patients as our institution began measuring lactate on septic patients fairly recently. As such, the sample size was small and there was no control group to compare mortality and outcomes with.

## Conclusion

This is a pilot study and the aim was to look at an in depth analysis of sepsis in the specific dialysis population and examining the influence of fluid resuscitation, role of SIRS criteria and vasopressor use on their mortality. Hopefully, it will stimulate further prospective research trials focusing on sepsis in the ESRD population.

### Ethics committee

Manuscript was approved by the American University of Beirut Institutional Review Board Committee.

## Abbreviations

ED: Emergency department
ICU: Intensive care unit
GPU: General practice unit
SIRS: Systemic inflammatory response syndrome
EGDT: Early goal directed therapy
ESRD: End Stage Renal Disease (ESRD)
HD: Hemodialysis
